# Stability-instability transition in tripartite merged ecological networks

**DOI:** 10.1007/s00285-022-01783-7

**Published:** 2022-08-12

**Authors:** Clive Emary, Anne-Kathleen Malchow

**Affiliations:** 1 School of Mathematics, Statistics and Physics, Newcastle University, Newcastle-upon-Tyne, NE1 7RU UK; 2 Institute for Biochemistry and Biology, University of Potsdam, 14476 Potsdam, Germany

**Keywords:** Random matrices, Phase transition, Random eigenvalues, Population dynamics, Community matrix, Ecological network, 92D40, 60B20, 37H30

## Abstract

Although ecological networks are typically constructed based on a single type of interaction, e.g. trophic interactions in a food web, a more complete picture of ecosystem composition and functioning arises from merging networks of multiple interaction types. In this work, we consider tripartite networks constructed by merging two bipartite networks, one mutualistic and one antagonistic. Taking the interactions within each sub-network to be distributed randomly, we consider the stability of the dynamics of the network based on the spectrum of its community matrix. In the asymptotic limit of a large number of species, we show that the spectrum undergoes an eigenvalue phase transition, which leads to an abrupt destabilisation of the network as the ratio of mutualists to antagonists is increased. We also derive results that show how this transition is manifest in networks of finite size, as well as when disorder is introduced in the segregation of the two interaction types. Our random-matrix results will serve as a baseline for understanding the behaviour of merged networks with more realistic structures and/or more detailed dynamics.

## Introduction

A central goal of community ecology is to identify the mechanisms that maintain biodiversity in natural communities. One way to approach this problem is through the lens of ecological networks — abstract representations of interactions between taxa in a community (Montoya et al. 2006; Landi et al. 2018; Delmas et al. 2019). Classically, studies of ecological networks have tended to focus on a single type of interaction, e.g. trophic interactions in a food web (Pimm 1982; Dunne et al. 2002; Dunne 2012) or mutualistic interactions in a plant-pollinator network (Bascompte and Jordano 2013; Valdovinos 2019). Over the last decade or so, however, there has been a growing recognition that further progress requires the construction and study of networks that contain multiple interaction types (Ings et al. 2009; Fontaine et al. 2011; García-Callejas et al. 2018) and even combining ecological with social interactions (Felipe-Lucia et al. 2021).

**Fig. 1:**
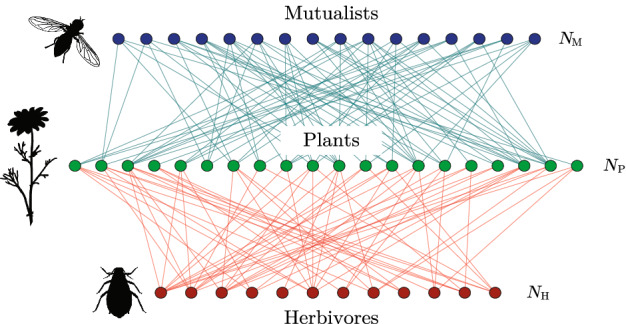
A tripartite ecological network consisting of $N_\mathrm {H}$ herbivores and $N_\mathrm {M}$ mutualists interacting with a set of $N_\mathrm {P}$ plants. Without disorder, all interactions between herbivores and plants are antagonistic (red edges), whereas all interactions between mutualists and plants are mutualistic (blue). Taxa images are public domain from http://www.phylopic.org (colour figure online)

In this paper we consider the stability of ecological networks based on the tripartite structure of Fig. 1. These networks consist of three guilds (nominally plants, herbivores and mutualists) interacting through two distinct sets of interactions, one antagonistic and the other mutualistic. This tripartite structure represents a minimal example of a merged network (Fontaine et al. 2011; Sauve et al. 2014), and reflects the large-scale structure seen in empirical networks such as those discussed by Melián et al. (2009), Pocock et al. (2012), Kéfi et al. (2016), Sauve et al. (2016), Miller et al. (2021), Laha et al. (2022). In the framework of multi-layer networks (Pilosof et al. 2017), Fig. 1 could be viewed as a two-layer multiplex network with antagonists in one layer, mutualists in the other and plants common to both. Two recent surveys analysed structural features and robustness of a number of empirical tripartite networks from the literature, including those with mutualist and antagonist partitions as considered here; Timóteo et al. (2021) found that the importance of a species is positively correlated between the two bipartite subnetworks, and Domínguez-García and Kéfi (2021) found that the robustness (with respect to plant losses) of tripartite mutualist-antagonist networks could be understood in terms of the robustnesses of the two bipartite networks composing them.

The aim of the present contribution is to provide an analysis of the dynamical stability of this network structure when the subnetwork interactions are described by random matrices. The use of random matrices to shed light on ecosystem stability has a long history (May 1972, 1974) and acts as a baseline scenario against which more detailed and ecologically-motivated studies can be compared (Allesina and Tang 2012). The stability of networks comprising a mixture of antagonistic and mututalistic interactions has previously been considered from a random-matrix viewpoint (Mougi and Kondoh 2012; Suweis et al. 2014; Sellman et al. 2016). However, these studies have been of (stochastically) homogeneous models, i.e. without the tripartite structure of Fig. 1. The persistence and stability of an ecosystem with this structure was considered in Sauve et al. (2014) using numerical simulations for small networks. In contrast, the focus of our work is on analytic results for ecosystems consisting of a large number of species.

Specifically we investigate the stability of a community matrix with block-structure that reflects Fig. 1 and with random interactions within the blocks. Using the results of Marčenko and Pastur (1967) and Benaych-Georges and Nadakuditi (2011) we give an account of key spectral features of such matrices in the limit of large network size. We show that the spectrum consists of a bulk component and a pair of eigenvalues of large magnitude. The properties of these latter split the behaviour of the model into two distinct phases. In the antagonist-dominated phase, the large eigenvalues are complex and the stability properties of the model are determined by the bulk. In the mutualist-dominated phase, the large eigenvalues are real and serve to destabilise the system. The transition between these two phases represents an eigenvalue phase transition (Baik et al. 2005) and can be driven, for example, by an increase in the relative fraction of mutualists in the ecosystem. Whilst this phase transition strictly takes place in the asymptotic limit, we also derive an expression for the stability-determining eigenvalue valid at finite system size. Finally, we also consider a scenario in which we introduce a degree of disorder into the network of Fig. 1 by exchanging the type of a certain random fraction of interactions. We show that the phase transition can survive in the presence of disorder, but vanishes if the disorder is too strong.

## Tripartite network model

We consider a system of $N_\mathrm {P}$ plants (or producers), $N_\mathrm {M}$ mutualists and $N_\mathrm {M}$ herbivores such that the total number of consumers (mutualists and herbivores) is $N_\mathrm {C} = N_\mathrm {M}+N_\mathrm {H}$. We then define $s={N_\mathrm {C}}/{N_\mathrm {P}}$ as the ratio of consumers to plants and $r={N_\mathrm {M}}/{N_\mathrm {C}}$ as the ratio of mutualists to consumers. We assume that, close to equilibrium, the dynamics of the populations is described by community matrix $K=-D + A$, where *D* describes intraspecific competition and *A* describes interspecific interactions. As is conventional, we assume *D* to have elements $D_{ij} = d \delta _{ij}$ with $\delta _{ij}$ the Kronecker delta symbol and *d* the competition strength. We then choose *K* to reflect the tripartite structure of the network in Fig. 1. In the first instance, we take *A* to have the following block structure

1 $$\begin{aligned} A = \frac{1}{\sqrt{N_\mathrm {P}}} \begin{pmatrix} 0 &{} M &{} -H \\ \mu _M M^T &{} 0 &{} 0 \\ \mu _H H^T &{} 0 &{} 0 \end{pmatrix} , \end{aligned}$$

in which the species have been ordered plants first, then mutualists and finally herbivores. The block *M* is of dimension $N_\mathrm {P} \times N_\mathrm {M}$ and describes the plant-mutualist interactions; block *H* is of dimension $N_\mathrm {P} \times N_\mathrm {H}$ and describes plant-herbivore interactions. We take all matrix elements of *M* and *H* to be positive such that the nature of the interactions is encoded in the explicit signs shown in Eq. (1). Parameters $\mu _M$ and $\mu _H$ are included to describe asymmetries in the two directions of each interaction type. The forefactor $N_\mathrm {P}^{-1/2}$ is included for mathematical convenience. We note that a similar matrix block structure was considered by Johnson et al. (2014) but with sign assignments appropriate to exclusively trophic interactions.

This overall structure is then supplemented by a random model for the matrix elements of the blocks. Considering the mutualists, we set $M_{ij} = e_{ij}^\mathrm {M} m_{ij}$ where the interaction coefficients $m_{ij}$ are treated as independent identical random variables with mean ${\overline{m}}$ and variance $\sigma _m^2$, and where the factors $e^\mathrm {M}_{ij}$ describe the links of the plant-mutualist sub-network: species that interact have $e^\mathrm {M}_{ij}=1$; those that do not have $e^\mathrm {M}_{ij}=0$. The mutualist subnetwork connectance is therefore $c_M = \sum _{i=1}^{N_\mathrm {P}}\sum _{j=1}^{N_\mathrm {M}} e^\mathrm {M}_{ij}/(N_\mathrm {M} N_\mathrm {P})$. We assume a random structure for the plant-mutualist network with $e^\mathrm {M}_{ij}$ chosen from a Bernoulli distribution with probability $P(e^\mathrm {M}_{ij}=1)=c_M$ for all pairs *i*, *j*. The first and second cumulants of the matrix elements of *M* are thus

2 $$\begin{aligned} \langle [M] \rangle = c_M{\overline{m}} \quad \text {and}\quad \langle [M]^2 \rangle _c = \sigma _M^2 = c_M \left[ \sigma ^2_m + (1-c_M){\overline{m}}^2\right] . \end{aligned}$$

An analogous set-up and notation is employed for the matrix elements of *H*.

## Eigenvalue spectrum

The stability of the equilibrium described by community matrix *K* is determined by its $N_\mathrm {C}+N_\mathrm {P}$ eigenvalues, $\epsilon$, which obey $K {\mathbf {v}} = \epsilon {\mathbf {v}}$. Writing eigenvector ${\mathbf {v}}$ in terms of three vectors, one for each component of the system, ${\mathbf {v}} = ({\mathbf {v}}_\mathrm {P},{\mathbf {v}}_\mathrm {M},{\mathbf {v}}_\mathrm {H})$ and taking the block structure of *K* into account we arrive at the set of three equations

3 $$\begin{aligned} N_\mathrm {P}^{-1/2} \left( M {\mathbf {v}}_\mathrm {M} - H {\mathbf {v}}_\mathrm {H} \right)= & {} \left( \epsilon + d \right) {\mathbf {v}}_\mathrm {P} ;\qquad \quad \quad \nonumber \\ N_\mathrm {P}^{-1/2} \mu _\mathrm {M} M^T {\mathbf {v}}_\mathrm {P}= & {} \left( \epsilon + d \right) {\mathbf {v}}_\mathrm {M} ; \qquad N_\mathrm {P}^{-1/2} \mu _\mathrm {H} H^T {\mathbf {v}}_\mathrm {P} =\left( \epsilon + d \right) {\mathbf {v}}_\mathrm {H}. \end{aligned}$$

Eliminating ${\mathbf {v}}_\mathrm {M}$ and ${\mathbf {v}}_\mathrm {H}$ from the first of these, we obtain the eigenvalue equation

4 $$\begin{aligned} {\mathcal {W}} {\mathbf {v}}_P = \lambda {\mathbf {v}}_P , \end{aligned}$$

in which

5 $$\begin{aligned} {\mathcal {W}} = N_\mathrm {P}^{-1} \left( M M^T - \gamma H H^T \right) , \end{aligned}$$

$\lambda = \left( \epsilon +d^2 \right) /\mu _\mathrm {M}$ and $\gamma = \mu _H/\mu _M$. The stability of community matrix *K* therefore becomes a question of the spectrum of ${\mathcal {W}}$ with the eigenvalues of *K* related to those of ${\mathcal {W}}$ through^1^

6 $$\begin{aligned} \epsilon = -d \pm \sqrt{\mu _M \lambda } . \end{aligned}$$

Accordingly, each positive eigenvalue $\lambda > 0$ contributes two real eigenvalues $\epsilon$ to the spectrum of *K*, whereas a negative eigenvalue $\lambda < 0$ contributes an imaginary pair to the spectrum of *K*. The number of zero eigenvalues of *K* depends on *s*, the ratio of consumers to plants. For $s>1$, i.e. more consumers than plants, matrix ${\mathcal {W}}$ is of full rank and thus has $N_P$ non-zero eigenvalues $\lambda \ne 0$, equating to $2 N_P$ non-zero eigenvalues for *K*. Matrix *K* then has an additional $N_C-N_P$ zero eigenvalues. On the other hand, if $s<1$, ${\mathcal {W}}$ is rank deficient and has only $N_C$ non-zero eigenvalues. Matrix *K* then has a total of $N_P-N_C$ zero eigenvalues.

**Fig. 2:**
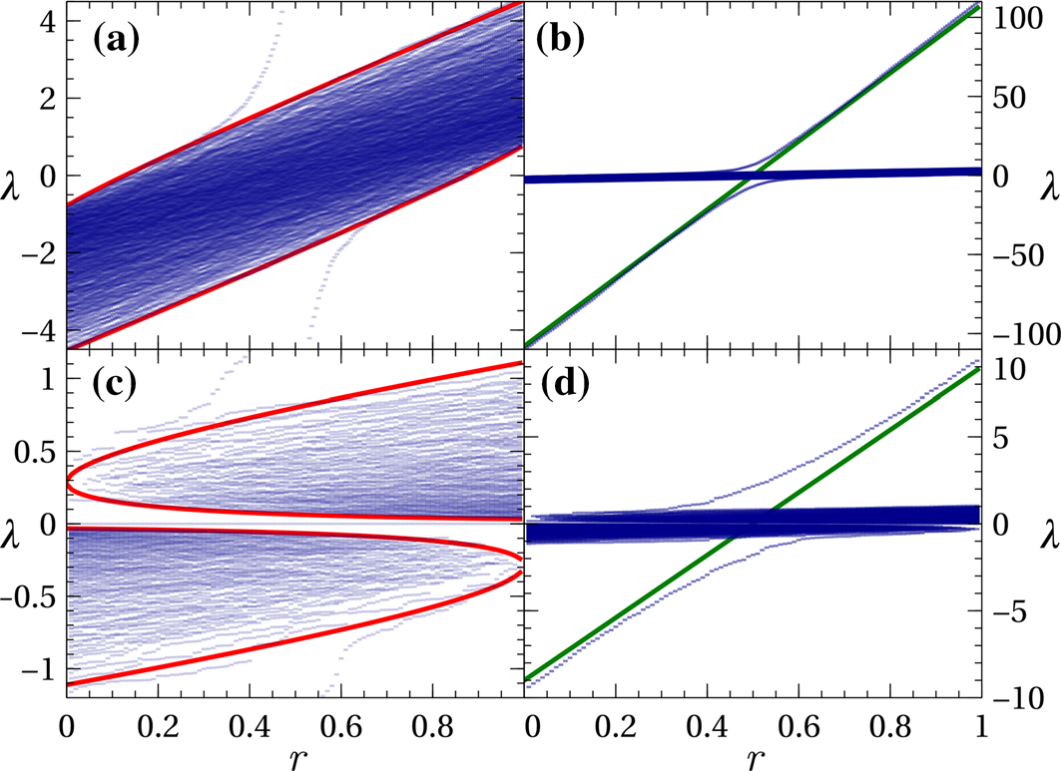
The spectrum of matrix ${\mathcal {W}}$ as a function of mutualist ratio *r*. Panels (a) and (b) shows results for a high ratio of consumers to plants, $s = 6$; (c) and (d) show results for a small ratio, $s=1/2$. The lefthand panels show a close up of the bulk part of the spectrum whilst the righthand panels show the same results on an expanded scale in which large eigenvalues above and below the bulk are readily apparent. Blue symbols are from numerical diagonalisation; red lines on the left are the analytic bulk spectrum edges from Eq. (18) and Eq. (19); green lines on the right are the asymptotic macroscopic value $N_\mathrm {P}\theta$ from Eq. (21). Parameters were $N_\mathrm {P}=200$, $c_M=c_H =0.3$, $\gamma =1$, with non-zero matrix elements chosen from half-normal distributions with ${\overline{m}}={\overline{h}}=1$ (colour figure online)

The main features in the spectrum of ${\mathcal {W}}$ can be appreciated from numerical diagonalisation. For concreteness, we draw the nonzero elements of *M* from a half-normal distribution with probability density function

7 $$\begin{aligned} f(m) = 2/(\pi {\overline{m}}) \exp \left[ -m^2/\left( \pi {\overline{m}}^2 \right) \right] , \qquad \text {for}\qquad m\ge 0. \end{aligned}$$

The standard deviation of this distribution is $\sigma _m = {\overline{m}}\sqrt{\pi /2-1}$. The nonzero elements of *H* are generated in a similar fashion but using parameter ${\overline{h}}$. Figure 2 shows the numerical spectrum of instances of matrix ${\mathcal {W}}$ across the range of values of the mutualist ratio *r* with $N_\mathrm {P}$ and $N_\mathrm {C}$ fixed. For $s>1$ [Figs. 2a and b] the spectrum clearly separates into a compact “bulk” spectrum located around $\lambda =0$, plus up to two large eigenvalues situated outside the bulk with magnitudes strongly dependent on *r*. The situation for $s<1$ [Figs. 2c and d] is similar, except that here the single bulk is replaced by two disjoint lobes with a further collection of eigenvalues of zero. The large eigenvalues are equally apparent in this case.

## Eigenvalue phase transition

We now consider the spectrum of ${\mathcal {W}}$ analytically and show that the largest eigenvalue undergoes an eigenvalue phase transition in the $N_\mathrm {P}\rightarrow \infty$ limit with the ratios *s* and *r* held fixed. We begin by writing ${\mathcal {W}} = {\mathcal {B}} + {\mathcal {P}}$ with

8 $$\begin{aligned} {\mathcal {B}} = N_\mathrm {P}^{-1} \mathcal {X T X}^T, \end{aligned}$$

where matrix ${\mathcal {X}}$ has dimensions $N_\mathrm {P} \times N_\mathrm {C}$ and elements

9 $$\begin{aligned} {\mathcal {X}}_{ij} = {\left\{ \begin{array}{ll} \left( M_{ij} - \langle [M] \rangle \right) / \sigma _M &{} 1 \le j \le N_\mathrm {M},\\ \left( H_{ij} - \langle [H] \rangle \right) / \sigma _H &{} N_\mathrm {M}+1 \le j \le N_\mathrm {C}, \end{array}\right. } \end{aligned}$$

and with ${\mathcal {T}}=\mathrm {diag}\left( \tau _1,\tau _2,\ldots \tau _{N_\mathrm {C}} \right)$ with

10 $$\begin{aligned} \tau _{i} = {\left\{ \begin{array}{ll} \tau _\mathrm {M}=\sigma _M^2 &{} 1 \le i \le N_\mathrm {M},\\ \tau _\mathrm {H}=-\gamma \sigma _H^2 &{} N_\mathrm {M}+1 \le i \le N_\mathrm {C}. \end{array}\right. } \end{aligned}$$

The second matrix in the decomposition of ${\mathcal {W}}$ reads

11 $$\begin{aligned} {\mathcal {P}}_{ij}= & {} \theta + \frac{1}{N_\mathrm {P}}\sum _{k=1}^{N_\mathrm {C}} \kappa _k \left( x_{ik}+x_{jk} \right) , \end{aligned}$$

with

12 $$\begin{aligned} \theta= & {} s \left\{ r \langle [M] \rangle ^2 - \gamma (1-r) \langle [H] \rangle ^2\right\} , \end{aligned}$$

and

13 $$\begin{aligned} \kappa _{i} = {\left\{ \begin{array}{ll} \langle [M] \rangle \sigma _M &{} 1 \le i \le N_\mathrm {M},\\ -\gamma \langle [H] \rangle \sigma _H &{} N_\mathrm {M}+1 \le i \le N_\mathrm {C} . \end{array}\right. } \end{aligned}$$

The elements of matrix ${\mathcal {X}}$ are independent random variables each with zero mean and unit variance. Moments beyond the second play no role in our results in the large-$N_\mathrm {P}$ limit, and thus we take all ${\mathcal {X}}_{ij}$ to be distributed identically. Turning to Eq. (11), we observe that the second term is a summation over a large number of independent variables, and thus gives a contribution of the order $N_\mathrm {C}^{1/2}$ for large $N_\mathrm {C}$. Overall, then, this term scales like $N_\mathrm {P}^{-1/2}$ and is therefore negligible in comparison with the first term which scales like $\theta \sim 1$. Thus, for ${\mathcal {P}}$, we obtain the approximate rank-1 form

14 $$\begin{aligned} {\mathcal {P}} \sim \theta \, \mathbb {J} \end{aligned}$$

with $\mathbb {J}$ the $N_\mathrm {P}\times N_\mathrm {P}$ matrix of ones.

We first consider the matrix ${\mathcal {B}}$ by itself. Let its ordered eigenvalues be $\lambda _{1}({\mathcal {B}}) \ge \cdots \ge \lambda _{N_\mathrm {P}}({\mathcal {B}})$ and define the probability measure $\mu _{{\mathcal {B}}}$ as the limiting empirical eigenvalue distribution $\mu _{{\mathcal {B}}} = \lim _{N_\mathrm {P} \rightarrow \infty } N_\mathrm {P}^{-1} \sum _{j=1}^{N_\mathrm {P}} \delta _{\lambda _{j}({\mathcal {B}})}$. From Marčenko and Pastur (1967) [see also Silverstein and Bai (1995)], the Stieltjes transform of $\mu _{\mathcal {B}}$ is given by

15 $$\begin{aligned} G_{\mu _{\mathcal {B}}}(z) = \int \frac{1}{z-t} d \mu _{\mathcal {B}}(t) = \left[ z - \int \frac{s\tau d\nu (\tau )}{1-\tau G_{\mu _{\mathcal {B}}}(z)}\right] ^{-1} , \end{aligned}$$

where $\nu (\tau )$ is the $N_\mathrm {P} \rightarrow \infty$ probability distribution function of the values $\left\{ \tau _1, \ldots , \tau _{N_\mathrm {C}} \right\}$. For the case in hand, this reads

16 $$\begin{aligned} d\nu (\tau ) = \left[ r \delta (\tau -\tau _\mathrm {M}) + (1-r) \delta (\tau -\tau _\mathrm {H}) \right] d\tau , \end{aligned}$$

and thus we obtain

17 $$\begin{aligned} G_{\mu _{\mathcal {B}}}(z) = \left[ z - \frac{r s\tau _\mathrm {M}}{1-\tau _\mathrm {M} G_{\mu _{\mathcal {B}}}(z)} - \frac{(1-r)s\tau _\mathrm {H}}{1-\tau _\mathrm {H} G_{\mu _{\mathcal {B}}}(z)} \right] ^{-1} , \end{aligned}$$

and its inverse

18 $$\begin{aligned} z_{\mu _{\mathcal {B}}}(G) = \frac{1}{G} + \int \frac{s\tau d\nu (\tau )}{1-\tau G} = \frac{1}{G} + \frac{r s\tau _\mathrm {M}}{1-\tau _\mathrm {M} G} + \frac{(1-r) s\tau _\mathrm {H}}{1-\tau _\mathrm {H} G} \end{aligned}$$

Again from Marčenko and Pastur (1967), the edges of connected components of spectrum can be obtained as $b = z_{\mu _{\mathcal {B}}}(G_b)$ where $G_b$ is a solution of

19 $$\begin{aligned} \left. \frac{dz_{\mu _{\mathcal {B}}}}{dG}\right| _{G=G_b} = -\frac{1}{{G_b}^2} + \frac{r s\tau _\mathrm {M}^2}{(1-\tau _\mathrm {M} G_b)^2} + \frac{(1-r) s\tau _\mathrm {H}^2}{(1-\tau _\mathrm {H} G_b)^2} =0 . \end{aligned}$$

In particular, this gives us $b_+$ and $b_-$ the upper and lower edges of the bulk part of the spectrum, i.e. the supremum and infimum of the support of $\mu _{\mathcal {B}}$. Figure 2 shows the boundaries obtained from Eq. (19) superimposed on the numerical spectra. The boundaries, including $b_\pm$, give good approximation to the numerical bulk edges across the entire range of *r*.

Now consider the complete matrix, ${\mathcal {W}} = {\mathcal {B}} + {\mathcal {P}}$. Applying Theorem 2.1 of Benaych-Georges and Nadakuditi (2011) to the rank-1 perturbation of Eq. (14), we have that in the $N_\mathrm {P}\rightarrow \infty$ limit the uppermost eigenvalue of ${\mathcal {W}}$ almost surely converges as

20 $$\begin{aligned} \lambda _1({\mathcal {W}}) \rightarrow {\left\{ \begin{array}{ll} z_{\mu _{\mathcal {B}}}\left[ (N_\mathrm {P}\theta )^{-1}\right] &{} \text { if } N_\mathrm {P}\theta >1/G_{\mu _{\mathcal {B}}}(b_+), \\ b_+ &{}\text {otherwise.} \end{array}\right. } \end{aligned}$$

Given that $z_{\mu _{\mathcal {B}}}$ is a monotonically decreasing function at the spectrum edge and that $N_\mathrm {P}$ is large, this becomes

21 $$\begin{aligned} \lambda _1({\mathcal {W}}) \rightarrow \mathrm {max}[b_+,N_\mathrm {P}\theta ] . \end{aligned}$$

Thus, in the $N_\mathrm {P}\rightarrow \infty$ limit, the largest eigenvalue of ${\mathcal {W}}$ undergoes an abrupt eigenvalue phase transition (Baik et al. 2005) from a value $b_+\sim (N_\mathrm {P})^0$ below the transition to a value $\lambda _1 = N_\mathrm {P} \theta$ that is “macroscopic” in the sense that it scales with the size of the ecosystem. This transition occurs at $N_\mathrm {P} \theta = b_+$ but given the different scaling of the two sides of this equation, the transition point is effectively given by $\theta = 0$ in the asymptotic limit. Thus, we see that the phase transition occurs when $r=r_\star$ with the critical mutualist ratio

22 $$\begin{aligned} r_\star = \frac{\gamma \langle [H] \rangle ^2}{\langle [M] \rangle ^2 + \gamma \langle [H] \rangle ^2} . \end{aligned}$$

The significance of this is that eigenvalue $\lambda _1({\mathcal {W}})$ determines the stability of community matrix *K* and is thus an order parameter for a phase transition in the stability of the model. We note that the quantity $N_\mathrm {P} \theta$ is the row sum of matrix ${\mathcal {W}}$, as previously discussed by Allesina and Tang (2012) in the context of isolated eigenvalues of mutualist matrices. Considering the lowest eigenvalue, we similarly find $\lambda _{N_\mathrm {P}}({\mathcal {W}}) = \mathrm {min}[b_-,N_\mathrm {P}\theta ]$. This also exhibits a phase transition but this is of little significance from a point of view of the stability.

Figure 2 shows the macroscopic value $N_\mathrm {P}\theta$ superimposed on the numerical spectra, where it is seen to be close to numerically-obtained large eigenvalues when they lie significantly outside the bulk. Deviations from this good agreement occur in the region close to where $N_\mathrm {P}\theta$ and $b_\pm$ cross, and this is a consequence of the finite value of $N_\mathrm {P}$ in this figure.

Finally we note that there is a secondary, less dramatic stability transition implicit in the above results and visible in Fig. 2. This transition occurs at the point $b_+=0$. Below this point, the spectrum of ${\mathcal {W}}$ is completely negative [see for example the lefthand edge of Fig. 2a] and thus $\sqrt{\mu _M \lambda }$ will be imaginary for all eigenvalues. The stability of community matrix *K* will, from Eq. (6), then be given purely by the intraspecific competition term. Above this point, $\sqrt{\mu _M \lambda }$ will be real for some $\lambda$ and this will then start to reduce the stability.

## Finite-size expression for the large eigenvalue

Whilst the above captures the emergence of a macroscopic eigenvalue, Fig. 2 shows that at finite $N_\mathrm {P}$ the uppermost eigenvalue goes smoothly over from the bulk edge at low *r* to the macroscopic disjoint value at high *r*. In this section we derive an expression for $\lambda _1$ that captures this behaviour at large but finite $N_\mathrm {P}$. In doing so, we consider a slightly more general model, namely

23 $$\begin{aligned} A = \frac{1}{\sqrt{N_\mathrm {P}}} \begin{pmatrix} 0 &{} M_1 &{} -H_1 \\ \mu _M M_2^T &{} 0 &{} 0 \\ \mu _H H_2^T &{} 0 &{} 0 \end{pmatrix} , \end{aligned}$$

where $M_1$ and $M_2$ are random blocks with relationship left unspecified, and similarly for $H_1$ and $H_2$. This generalisation allows us to discuss the effect of disorder in the next section. Clearly the model of the previous section is recovered by setting $M_1=M_2=M$ and $H_1=H_2=H$.

With the interaction matrix of Eq. (23), the $N_\mathrm {P}\times N_\mathrm {P}$ matrix equivalent of Eq. (5) is

24 $$\begin{aligned} {\mathcal {W}} = N_\mathrm {P}^{-1}\left( M_1 M_2^T - \gamma H_1 H_2^T \right) . \end{aligned}$$

An approximate account of the spectrum of ${\mathcal {W}}$ can be obtained by considering this matrix in the eigenbasis of its ensemble average $\langle W \rangle$. Doing so allows us to identify the scaling properties of different parts of the matrix and derive an approximate expression valid for $N_\mathrm {P} \gg 1$. Details of this calculation are described in the Appendix with the results as follows. The spectrum of ${\mathcal {W}}$ is seen to approximately contain $N_\mathrm {P}-2$ eigenvalues at

25 $$\begin{aligned} \phi= & {} s \left\{ r \langle [M_1][M_2] \rangle _c - (1-r) \gamma \langle [H_1][H_2] \rangle _c \right\} , \end{aligned}$$

in which $\langle [M_1][M_2] \rangle _c$ is the covariance between the matrix elements of $M_1$ and $M_2$ (and similarly for $\langle [H_1][H_2] \rangle _c$). This set of eigenvalues is the approximate representation of the bulk in this calculation. More importantly, we also obtain two non-trivial eigenvalues given by

26 $$\begin{aligned} \lambda _\pm = \frac{N_\mathrm {P}}{2} \left\{ \theta + \frac{2 \phi }{N_\mathrm {P}} \pm \sqrt{ \theta ^2 + \frac{\varOmega ^2}{N_\mathrm {P}} } \right\} , \end{aligned}$$

in which, similar to Eq. (12), we have

27 $$\begin{aligned} \theta= & {} s \left\{ r \langle [M_1] \rangle \langle [M_2] \rangle - \gamma (1-r) \langle [H_1] \rangle \langle [H_2] \rangle \right\} , \end{aligned}$$

and where

28 $$\begin{aligned} \varOmega ^2= & {} 4 s \left\{ r\langle [M_1][M_2] \rangle _c \langle [M_1] \rangle \langle [M_2] \rangle + (1-r)\langle [H_1][H_2] \rangle _c \langle [H_1] \rangle \langle [H_2] \rangle \right\} .\qquad \end{aligned}$$

In the asymptotic limit, Eq. (26) gives $\lambda _+ \rightarrow N_\mathrm {P} \theta$, which recovers the macroscopic eigenvalues obtained previously, and $\lambda _- \rightarrow \phi$ which then becomes part of the bulk.

**Fig. 3:**
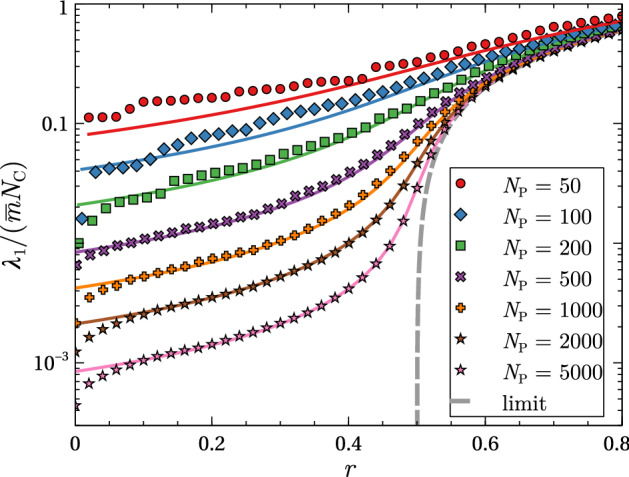
Largest eigenvalue $\lambda _1$ of matrix ${\mathcal {W}}$ as a function of the mutualist ratio *r* for several values of the plant number $N_\mathrm {P}$ and fixed $s=N_\mathrm {C}/N_\mathrm {P}=1/2$. Symbols show numerical results for a single random instances of ${\mathcal {W}}$; solid lines show the analytic result $\lambda _+$ of Eq. (26); the dashed line shows the asymptotic result of Eq. (21). Other parameters as in Fig. 2

Figure 3 compares the analytic expression $\lambda _+$ with the maximum eigenvalue extracted from numerical diagonalisation of ${\mathcal {W}}$ with $M_1=M_2=M$ and $H_1=H_2=H$ as in Fig. 2. Results for a range of different values of plant number $N_\mathrm {P}$ are shown. Clear agreement between numerical and analytic results is observed, with the degree of agreement increasing with $N_\mathrm {P}$ as expected. The only significant deviation between the two at large $N_\mathrm {P}$ occurs at very small values of *r*, where $\lambda _+$ overestimates $\lambda _1$. In this regime the scaling arguments leading to Eq. (26) break down.

One additional feature that is revealed by this finite-size analysis is the role of correlations between the strengths of the two directions of the interactions (Tang et al. 2014). The influence of these is manifest by the presence of the covariances $\langle [M_1][M_2] \rangle _c$ and $\langle [H_1][H_2] \rangle _c$ in $\langle \varOmega ^2 \rangle$. Since this term enters the expression for $\lambda _\pm$ with a $N_\mathrm {P}^{-1}$ forefactor, correlations can only play a role at finite $N_\mathrm {P}$. In the maximally correlated case, we recover the model of Sect. 2 for which $\langle [M_1][M_2] \rangle _c = \langle [M]^2 \rangle _c$ as in Eq. (2) and and similarly for *H*. In a scenario where the interaction strengths are completely uncorrelated, we have $\langle [M_1][M_2] \rangle _c = \langle [H_1][H_2] \rangle _c = 0$ and $\langle \varOmega ^2 \rangle$ vanishes. The prediction for $\lambda _1$ in this case becomes the piecewise linear function $\lambda _1 = \mathrm {max}\left[ \phi ,\phi + N_\mathrm {P} \theta \right]$, similar to that found in Sect. 4 in the $N_\mathrm {P}\rightarrow \infty$ limit. Thus the “avoided crossing” that occurs in the correlated case at large but finite $N_P$ gives way to an actual crossing when the degree of correlation is zero.

## Interaction disorder

As defined by their interactions with plants, the consumers in Fig. 1 are either 100% mutualist or 100% herbivore. In this section we look what happens when we move away from this perfectly ordered scenario and swap the types of a random selection of the species interactions. We consider the same tripartite network as before, but with a probability $f^{\mathrm {M}}$ we swap the ($++$) signs of the mutualistic interactions to the ($+-$) signs of an antagonistic one. For the antagonistic interactions, we do the opposite with a probability $f^{\mathrm {H}}$. The end result is that the animal species no longer act with well defined roles, but differently across their connected plant species. Mathematically, this is incorporated into the framework of Sect. 5 by selecting the matrix elements of Eq. (23) to be

29 $$\begin{aligned} \left[ M_1 \right] _{ij}= & {} e_{ij}^\mathrm {M} \left[ (1-f^{\mathrm {M}}_{ij})m_{ij} - f^{\mathrm {M}}_{ij}h'_{ij} \right] \quad \left[ M_2 \right] _{ij} = e_{ij}^\mathrm {M} \left[ (1-f^{\mathrm {M}}_{ij})m_{ij} +\gamma f^{\mathrm {M}}_{ij}h'_{ij} \right] \nonumber \\ \left[ H_1 \right] _{ij}= & {} e_{ij}^\mathrm {H} \left[ (1-f^{\mathrm {H}}_{ij})h_{ij} - f^{\mathrm {H}}_{ij}m'_{ij} \right] \quad \left[ H_2 \right] _{ij} = e_{ij}^\mathrm {H} \left[ (1-f^{\mathrm {H}}_{ij})h_{ij} +\gamma ^{-1} f^{\mathrm {H}}_{ij}m_{ij}' \right] .\nonumber \\ \end{aligned}$$

Here, $m_{ij}$ and $h_{ij}$ are random variables as before, $m'_{ij}$ and $h'_{ij}$ are further independent random variables chosen from the same distributions, and $f_{ij}^{(M,H)}\in \{0,1\}$ are an additional set of binary random variables that describe whether interaction *ij* in block (*M*, *H*) is flipped or not. These latter are set with probability $P(f_{ij}^{(M,H)} = 1) = f^{(M,H)}$.

Using the results of the previous section, we see that, in the asymptotic limit, the largest eigenvalue of the matrix ${\mathcal {W}}$ is $\lambda _1 = \mathrm {max}[0,N_\mathrm {P} \theta ]$ with $\theta$ of Eq. (27) in terms of the ensemble averages

30 $$\begin{aligned} \langle [M_1] \rangle= & {} c_M\left[ (1-f^{\mathrm {M}}){\overline{m}} - f^{\mathrm {M}} {\overline{h}} \right] \quad \langle [M_2] \rangle = c_M\left[ (1-f^{\mathrm {M}}){\overline{m}} + \gamma f^{\mathrm {M}} {\overline{h}} \right] \nonumber \\ \langle [H_1] \rangle= & {} c_H\left[ (1-f^{\mathrm {H}}){\overline{h}} - f^{\mathrm {H}} {\overline{m}} \right] \quad \langle [H_2] \rangle = c_H\left[ (1-f^{\mathrm {H}}){\overline{h}} + \gamma ^{-1} f^{\mathrm {H}} {\overline{m}} \right] .\qquad \end{aligned}$$

**Fig. 4:**
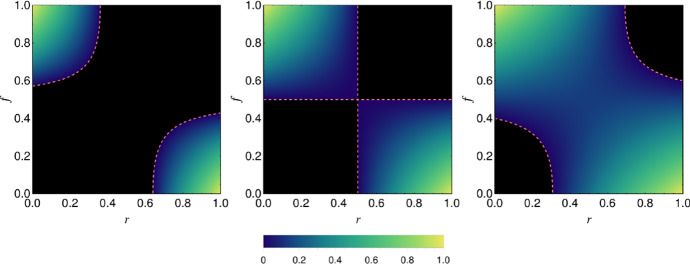
Phase diagrams for the tripartite network of Fig. 1 in which a random fraction *f* of interactions have their type (mutualistic or antagonsitic) switched. Shown is the asymptotic order-parameter eigenvalue $\lambda _1 =\mathrm {max}[0,N_\mathrm {P} \theta ]$ with $\theta$ of Eq. (27) evaluated with the mean values of Eq. (30) as a function of *f* and the mutualist ratio *r*. We have scaled $\lambda _1$ by its maximum value (obtained at $r=\gamma =1$ and $f=0$) and this removes the dependence on $N_\mathrm {P}$, *s*, and the connectance when $c_M=c_H$ as here. Results are shown for three values of the mutualist interaction strength: ${\overline{m}}= 0.75,1,1.5$ from left to right with ${\overline{h}}=1$ and $\gamma =1$. The black regions correspond to the antagonistic phase with $\lambda _1=0$ and the coloured regions, the mutualistic phase $\lambda _1>0$. The dashed line represents the phase boundary

Results are shown in Fig. 4 as phase diagrams in the plane defined by the mutualist ratio *r* and the fraction of swapped interactions $f^{\mathrm {M}} = f^{\mathrm {H}}=f$.

When there is exact symmetry between the interactions strength, i.e. ${\overline{m}}={\overline{h}}$ and $\gamma =1$ (Fig. 4 middle), the behaviour observed in the fully coherent case, $f=0$, is preserved at finite *f*, up to a fraction $f=1/2$. The transition still occurs at $r=1/2$ and the only change is that the size of order parameter in mutualist phase is diminished. At the point $f=1/2$ the dominant roles of the “antagonistic” and “mutualistic” species swap and the two phases reverse accordingly. Away from this exact symmetry we have more complicated behaviour. For ${\overline{m}}<{\overline{h}}$ (assuming $\gamma =1$) the mutualist phase occupies a diminished area of the phase diagram. For values of *f* close to 1/2 the system remains in the antagonist phase across the whole range of *r* and no instability transition takes place. In contrast, for ${\overline{m}}>{\overline{h}}$, it is the antagonist phase that is diminished and for *f* close to 1/2 the system remains in the mutualist phase for all *r*. Indeed, specifying for simplicity the case of $c_M=c_H$, $f^{\mathrm {M}} = f^{\mathrm {H}}=f$ and $\gamma =1$ (as in Fig. 4), we find the value of *r* for which $\theta =0$ to be

31 $$\begin{aligned} r_\star = \frac{ {\overline{h}}^2(1-f)^2 - {\overline{m}}^2f^2 }{ \left( {\overline{h}}^2 + {\overline{m}}^2 \right) \left( 1-2 f \right) } . \end{aligned}$$

For a phase transition to occur, we require that $0< r_\star < 1$, which gives

32 $$\begin{aligned} \frac{f}{1-f}<\frac{{\overline{h}}}{{\overline{m}}}<\frac{1-f}{f} , \end{aligned}$$

as the condition on the interaction and disorder strengths for the existence of a phase transition.

## Discussion

We have studied the stability of a community matrix with random elements organised according to the tripartite structure of Fig. 1. This structure can be viewed as the mergence of two bipartite networks with changes in the mutualist ratio *r* interpolating between them. At $r=0$ we have a bipartite predator-prey model, whose community matrix has purely imaginary eigenvalues (Johnson et al. 2014); at $r=1$ we have a bipartite mutualist model, whose stability is determined by a single large real eigenvalue (Allesina and Tang 2012). We have shown here that the emergence of this macroscopic eigenvalue as a function of *r* is abrupt in the asymptotic limit, and that this represents an eigenvalue phase transition that takes place when *r* reaches the critical value $r_\star$. The behaviour of the model is thus split into two distinct regimes. For $r< r_\star$, we obtain an “antagonistic phase” in which the community matrix *K* can be stabilised by a small (i.e. order $N_\mathrm {P}^0$) value of the intraspecific competition *d*. In this regime, changing the number of mutualists by a small amount does not appreciably affect the stability of the system. In contrast, for $r>r_\star$ we obtain a “mutualist phase” in which stability is governed by the macroscopic eigenvalue such that the community matrix requires a large intraspecific competition, $d \sim N_\mathrm {P}^{1/2}$, to stabilise it. Moreover, in this phase, small changes in the mutualist number lead to large changes in $\lambda _1$ and hence dramatic changes in the stability of the system. Whilst we have framed this discussion in terms of the behaviour as a function of the ratio *r*, the phase transition can also be driven by changes in other parameters, e.g. mean interaction strengths or connectance. For example, setting $\theta =0$ for the ordered model of Sect. 2 shows that, with all other parameters fixed, the phase transition will occur when the ratio of the interaction strengths reaches a value

33 $$\begin{aligned} \left( \frac{{\bar{m}}}{{\bar{h}}} \right) ^2 = \gamma \frac{r}{1-r}\left( \frac{c_\mathrm {H}}{c_\mathrm {M}} \right) ^2 . \end{aligned}$$

A large positive eigenvalue of the community matrix is associated with the growth of populations away from the equilibrium values. The rapidity of growth seen here is a consequence of the positive feedback of “mutual benefaction” associated with the mutualistic interactions (Scheuring 1992). It should be born in mind, however, that the community matrix represents a linearisation of a more complex/detailed dynamical model. Thus, instability should not be interpreted as an uncontrolled growth, but rather as the indication of the shift of the ecosystem away from its equilibrium to a different one, the properties of which are outside the scope of the original model. This new equilibrium may well include fewer species than in the starting community.

The scaling in Eq. (1) is chosen such that the bulk spectrum of *K* converges in the $N_\mathrm {P}\rightarrow \infty$ limit. This follows from the convergence of the spectrum of ${\mathcal {W}}$ with bulk edges $b_\pm \sim (N_\mathrm {P})^0$. In the spectrum of *K*, this leaves the scaling of the macroscopic eigenvalue as $\epsilon = -d + \sqrt{\mu _M N_\mathrm {P}\theta } \sim \sqrt{N_\mathrm {P}}$. A slightly different choice would be to replace the $N_\mathrm {P}^{-1/2}$ forefactor out the front of Eq. (1) with $N_\mathrm {P}^{-1}$. This would make extreme eigenvalue of *K* converge to $\epsilon = -d + \sqrt{\mu _M \theta }\sim {N_\mathrm {P}}^0$ in the limit, whilst the bulk would shrink to a point at $\epsilon = -d$. Given the different scalings of the two parts of the spectrum, we might also consider a slightly different model in which this forefactor in Eq. (1) is omitted and we instead choose to scale the matrix-element distribution such that ${\overline{m}} \sim N_\mathrm {P}^{-1}$ and $\sigma _m \sim N_\mathrm {P}^{-1/2}$ and similarly for the herbivores. This situation would then be similar to the scaling in e.g. Bunin (2017), Galla (2018). This choice invalidates the assumptions upon which above calculations are based (operator ${\mathcal {P}}$ can no longer be approximated as above; the scaling arguments of Sect. 5 no longer hold). However, because here (and unlike references just cited) the matrix elements are strictly non-negative, $m_{ij},h_{ij}\ge 0$, the only way in which to obtain this scaling ${\overline{m}}$, ${\overline{h}}$, $\sigma _m$ and $\sigma _h$ is with a distribution that is concentrated at values of *m* extremely close to zero but which nevertheless has a very long tail. This scaling therefore converts most of the interactions in the community matrix into vanishing small ones (with a few very large ones to achieve the required mean) which seems rather at odds with the set-up of the model.

Phase transitions of the type described here have been described in related ecological models. Hogg et al. (1989) discussed an $S\times S$ community matrix with random elements distributed homogeneously about a finite mean, and showed that this can give rise to a macroscopic eigenvalue of size $\sqrt{S}$ (in the scaling convention pursued here). This model was also considered by Tang et al. (2014) and Tang and Allesina (2014) who described the emergence of a macroscopic eigenvalue as a phase transition from stability to instability as the mean value of the matrix elements is increased. The connection with the antagonist-mutualist system discussed here is that an increased mean interaction strength could arise from an increase in the number of mutualistic interactions. This scenario was discussed by Suweis et al. (2014) in another homogeneous model, introduced by Mougi and Kondoh (2012), that consists only of antagonists and mutualists. In these homogeneous models, the macroscopic eigenvalue can be understood in terms of the row sum of the community matrix *K* (Allesina and Tang 2012). In contrast, in the structured model discussed here, it is the row sum of the folded matrix ${\mathcal {W}}$ that is important.

Sauve et al. (2014) considered the same tripartite topology as discussed here. Their conclusion was that stability was enhanced by the connectance and species diversity of the mutualistic subnetwork but decreased by the connectance and diversity of the antagonistic one. This is opposite to the behaviour described here (where at best increased mutualistic interactions can leave the stability unaltered in the strict asymptotic limit). There are several reasons for this discrepancy. Sauve et al. (2014) consider a relatively small number of species (compared with the limiting behaviour here) described by a more detailed dynamical model with a particular subset of parameters chosen to guarantee that the mutualist subsystem was independently stable. Perhaps even more importantly is that the stability to which they refer is that of a final equilibrium state, obtained through the time-evolution of the model. This will in general have fewer species than the starting community and possess additional structure. It is also clear that non-random structure can affect the stability of such networks, as Sauve et al. (2016) show in their comparison of random and empirical networks.

It seems therefore that an important future direction is to look at the feasibility of dynamical systems with interaction topology similar to that in Fig. 1. This would also allow better connection with the persistence studies of Sauve et al. (2014) and functional extinctions of Sellman et al. (2016). The dynamical cavity method has proved very useful for studying feasibility in homogeneous models (Bunin 2017, Galla (2018)), and it remains on open possibility that this approach can be extended to structured models as studied here. Studying related few-species models, Ringel et al. (1996) commented that the destablizing effect of mutualisms “...is more than canceled by an increased chance of feasibility”, and it will be interesting to see whether this also applies for large networks of interacting species and, in particular, in the presence of a phase transition.

Finally we note that our analysis can be generalised to a more complex merged networks, reminiscent of that in Pocock et al. (2012), which consist of a central guild of $N_\mathrm {P}$ species (plants in Fig. 1) that interacts with a set of *G* other guilds, all of which are non-interacting with one another. Similar to Eq. (23), the interacting part of community matrix will be of the block form

34 $$\begin{aligned} A = \frac{1}{\sqrt{N_\mathrm {P}}} \begin{pmatrix} 0 &{} A_1 &{} A_2 &{} \cdots &{} A_G \\ B_1^T &{} 0 &{} 0 &{}\cdots &{} 0\\ B_2^T &{} 0 &{} 0 &{}\cdots &{} 0\\ \vdots &{} \vdots &{} \vdots &{} \ddots &{} \vdots \\ B_G^T &{} 0 &{} 0 &{}\cdots &{} 0 \end{pmatrix} , \end{aligned}$$

where $A_i$ and $B_i$ are random matrices of dimension $N_\mathrm {P} \times N_i$ with $N_i$ the number of species in guild *i*. In contrast to Eq. (23), the interaction signs [e.g. ($+$,−) for antagonisms] here are given by the matrix elements, rather than being explicit in the block structure. Similar to above, finding the spectrum of this *K* reduces to the problem of finding the spectrum of ${\mathcal {W}} = \sum _{i=1}^G A_i B_i^T$. This model possess a maroscopic eigenvalue, $\lambda = \sum _{i=1}^G N_i \langle \left[ A_i \right] \rangle \langle \left[ B_i \right] \rangle$ in the asymptotic limit, whose position relative to the bulk around zero determines the stability. From the interaction signs, a guild of mutualists gives a positive contribution to $\lambda$, whilst and antagonist give a negative one. Competitive interactions in which elements of both $A_i$ and $B_i$ are negative would then also give a positive contribution to $\lambda$, thus moving the system in the direction of instability.

## Finite-$N_\mathrm {P}$ expression for the largest eigenvalue

In this appendix we provide details on how the approximate expression for $\lambda _1$ in Sect. 5 is obtained. Our starting point is the more general model of Eq. (23) and the corresponding ${\mathcal {W}}$ matrix of Eq. (24). The ensemble average of ${\mathcal {W}}$ evaluates as

35 $$\begin{aligned} \langle {\mathcal {W}} \rangle = \phi \mathbbm {1} + \theta \mathbb {J} , \end{aligned}$$

with $\phi$ and $\theta$ as in Eq. (25) and Eq. (27) and where $\mathbbm {1}$ and $\mathbb {J}$ are the $N_\mathrm {P}\times N_\mathrm {P}$ unit matrix and matrix of ones, respectively. The eigenvalues of $\langle {\mathcal {W}} \rangle$ are $\phi + N_\mathrm {P}\theta$ and $\phi$, this latter being $(N_\mathrm {P}-1)$-fold degenerate. The eigenvector $u^{(1)}$ belonging to eigenvalue $\phi + N_\mathrm {P}\theta$ has elements

36 $$\begin{aligned} u^{(1)}_j= & {} \frac{1}{\sqrt{N_\mathrm {P}}};\qquad 1\le j \le N_\mathrm {P} . \end{aligned}$$

In the degenerate subspace, we choose the eigenvector set $\left\{ u^{(\alpha )}:2\le \alpha \le N_\mathrm {P}\right\}$ with elements

37 $$\begin{aligned} u^{(\alpha )}_j= & {} \sqrt{\frac{1}{N_\mathrm {P}(N_\mathrm {P}-1)}} \left( 1-N_\mathrm {P} \delta _{j,\alpha } \right) ;\qquad 1\le j \le N_\mathrm {P} . \end{aligned}$$

These states are normalised, orthogonal to $u^{(1)}$ but not orthogonal to each other. Indeed we have

38 $$\begin{aligned} u^{(\alpha )}\cdot u^{(\alpha )}= & {} 1 ;\qquad 2 \le \alpha \le N_\mathrm {P} \nonumber \\ u^{(1)}\cdot u^{(\beta )}= & {} 0 ;\quad \beta \ge 2 \nonumber \\ u^{(\alpha )}\cdot u^{(\beta )}= & {} \frac{1}{1-N_\mathrm {P}} ;\quad \alpha \ne \beta \ge 2 . \end{aligned}$$

These eigenvectors are easier to work with than orthogonalised ones, and since we work in the $N_\mathrm {P}$ limit, the effect of the finite overlap $u^{(\alpha )}\cdot u^{(\beta )}$ is negligible.

Arranging $u^{(\alpha )}$ as columns of matrix *U*, the full *W* matrix in this (ensemble-averaged) basis is ${\mathcal {V}} \equiv U^T {\mathcal {W}} U$, with matrix elements

39 $$\begin{aligned} {\mathcal {V}}_{11}= & {} u^{(1)}\cdot {\mathcal {W}} \cdot u^{(1)} = \frac{1}{N_\mathrm {P}} \sum _{ij} {\mathcal {W}}_{ij} ;\nonumber \\ {\mathcal {V}}_{1\beta }= & {} u^{(1)}\cdot {\mathcal {W}} \cdot u^{(\alpha )} = \frac{1}{N_\mathrm {P}\sqrt{N_\mathrm {P}-1}} \left\{ \sum _{ij} {\mathcal {W}}_{ij} - N_\mathrm {P} \sum _i {\mathcal {W}}_{i\beta } \right\} ; \nonumber \\ {\mathcal {V}}_{\alpha \beta }= & {} u^{(\alpha )}\cdot {\mathcal {W}} \cdot u^{(\beta )} = \frac{1}{N_\mathrm {P}(N_\mathrm {P}-1)} \left\{ \sum _{ij} {\mathcal {W}}_{ij} - N_\mathrm {P} \sum _i \left( {\mathcal {W}}_{i\beta } + {\mathcal {W}}_{\alpha i} \right) + N_\mathrm {P}^2{\mathcal {W}}_{\alpha \beta } \right\} ,\nonumber \\ \end{aligned}$$

in which indices $\alpha , \beta \ge 2$. Considering the scaling of the ensemble averages of these quantities, we find $\langle {\mathcal {V}} \rangle _{11} \sim N_\mathrm {P}$ , $\langle {\mathcal {V}} \rangle _{\alpha 1} = \langle {\mathcal {V}} \rangle _{1\beta } =0$, $\langle {\mathcal {V}} \rangle _{\alpha \alpha } \sim N_\mathrm {P}^0$, and $\langle {\mathcal {V}} \rangle _{\alpha \beta } \sim N_\mathrm {P}^{-1}$ for $\alpha \ne \beta$. Similarly, the variances scale as $\mathrm {Var}\left( {\mathcal {V}}_{11} \right) \sim N_\mathrm {P}^{0}$, $\mathrm {Var}\left( {\mathcal {V}}_{\alpha 1} \right) \sim \mathrm {Var}\left( {\mathcal {V}}_{1\beta } \right) \sim N_\mathrm {P}^{0}$, $\mathrm {Var}\left( {\mathcal {V}}_{\alpha \beta } \right) \sim N_\mathrm {P}^{-1}$. Thus, with $N_\mathrm {P}$ large, we drop ${\mathcal {V}}_{\alpha \beta }$ for $\alpha \ne \beta$ as being negligible, and replace the diagonal elements with their ensemble averages, as their fluctuations are negligible in comparison. Due to the vanishing of their mean, the elements ${\mathcal {V}}_{\alpha 1}$ and ${\mathcal {V}}_{1\beta }$ are left as is. Taken together, this gives the approximation

40 $$\begin{aligned} {\mathcal {V}} \approx \left( \begin{array}{cccc} \phi +N_\mathrm {P}\theta &{} {\mathcal {V}}_{12} &{} \ldots &{} {\mathcal {V}}_{1 N_\mathrm {P}}\\ {\mathcal {V}}_{12} &{} \phi &{} \ldots &{} 0 \\ \vdots &{} \vdots &{} \ddots &{} \vdots \\ {\mathcal {V}}_{1 N_\mathrm {P}} &{} 0 &{} \ldots &{} \phi \end{array} \right) . \end{aligned}$$

This can be diagonalised exactly. We obtain $N_\mathrm {P}-2$ eigenvalues of value $\phi$ and two nontrivial ones. These latter have the form of $\lambda _\pm$ from Eq. (26) with

41 $$\begin{aligned} \varOmega ^2 = \frac{4}{N_\mathrm {P}} \sum _{\alpha =2}^{N_\mathrm {P}} {\mathcal {V}}_{1\alpha }{\mathcal {V}}_{\alpha 1} = \frac{4}{N_\mathrm {P}^3} \left\{ N_\mathrm {P} \sum _{ijk} {\mathcal {W}}_{ij}{\mathcal {W}}_{jk} - \sum _{ijkl} {\mathcal {W}}_{ij}{\mathcal {W}}_{kl} \right\} . \end{aligned}$$

Evaluating the leading term in the ensemble average of $\varOmega ^2$ we find

42 $$\begin{aligned} \langle \varOmega ^2 \rangle\sim & {} 4 s \left\{ r\langle [M_1][M_2] \rangle _c \langle [M_1] \rangle \langle [M_2] \rangle + (1-r)\langle [H_1][H_2] \rangle _c \langle [H_1] \rangle \langle [H_2] \rangle \right\} \qquad \end{aligned}$$

A similar calculation for the variance show that it scales like $\mathrm {Var}\left( \varOmega ^2 \right) \sim N_\mathrm {P}^{-1}$ such that the fluctuations about the mean vanish in the limit. We thus approximate $\varOmega ^2$ with $\langle \varOmega ^2 \rangle$, thus giving the result stated in Eq. (28).

## Footnotes

1 A slightly expanded community matrix with different competition strengths for plants and consumers species ($d_P$ and $d_A$ respectively) has eigenvalues $\epsilon = \frac{1}{2}\left[ -d_A-d_P \pm \sqrt{\left( d_A-d_P \right) ^2 + 4\mu _M \lambda }\right]$ with the same $\lambda$.
